# Supplementary material for: "Why did our baby die soon after birth?"—Lessons on neonatal death in rural Cambodia from the perspective of caregivers
Source: PLoS One. 2021 Jun 7;16(6):e0252663. doi: 10.1371/journal.pone.0252663 (PMC8183999; doi:10.1371/journal.pone.0252663)
Supplement: S1 Checklist — (PDF) [file pone.0252663.s001.pdf]

S1 Checklist.
Consolidated criteria for reporting qualitative studies (COREQ): 32-item checklist

A checklist of items that should be included in reports of qualitative research. You must report the page number in your manuscript where you consider each of the items listed in this checklist. If you have not included this information, either revise your manuscript accordingly before submitting or note N/A.

No. Item	Guide questions/description	Reported on Page #/Response
**Domain 1: Research team and reflexivity**		
Personal Characteristics		
1. Interviewer/ facilitator	Which author/s conducted the interview or focus group?	**#6 (Data collection):**<br>25 trained surveyors conducted the interview under the supervision by one of the authors (AI).
2. Credentials	What were the researcher's credentials? E.g. PhD, MD	Not reported in the manuscript:<br>Researcher's credentials were medical doctor, midwife, nurse, pharmacist, or other bachelor holders. The detail is the next 3.Occupation.
3. Occupation	What was their occupation at the time of the study?	Not reported in the manuscript:<br>The surveyors consisted of two doctors, four nurses, three midwives, one pharmacist, seven statisticians, and eight sociologists.
4. Gender	Was the researcher male or female?	**#7 (Data collection):**<br>They were 14 females and 11 males.
5. Experience and training	What experience or training did the researcher have?	**#7 (Data collection):**<br>All surveyors had experience in conducting interview in the latest Cambodia Demographic Health Survey (CDHS) in 2014.
Relationship with participants		
6. Relationship established	Was a relationship established prior to study commencement?	Not reported in the manuscript:<br>No relationship was established between the study participants and the surveyors prior to the survey

7. Participant knowledge of the interviewer	What did the participants know about the researcher? e.g. personal goals, reasons for doing the research ?	**#8 (Ethical approval):**<br>The study objective was explained to all the participants, and written informed consent was obtained.
8. Interviewer characteristics	What characteristics were reported about the interviewer/facilitator? e.g. Bias, assumptions, reasons and interests in the research topic	**#7 (Data collection):**<br>All surveyors had experiences in conducting interviews using a standardized questionnaire, such as the Cambodia Demographic Health Survey. We also provided a training how to use the WHO standard verbal autopsy forms. Therefore, no bias was generated by the characteristics of surveyors.
**Domain 2: study design**		
Theoretical framework		
9. Methodological orientation and Theory	What methodological orientation was stated to underpin the study? e.g. grounded theory, discourse analysis, ethnography, phenomenology, content analysis	**#5 (Methods):**<br>Qualitative case study design was used in the analysis of verbal autopsy information.
Participant selection		
10. Sampling	How were participants selected? e.g. purposive, convenience, consecutive, snowball	**#6 (Data collection):**<br>Target villages were selected using two-stage cluster random sampling method. All households were involved in the study. Those who reported neonatal deaths participated in the study.
11. Method of approach	How were participants approached? e.g. face-to-face, telephone, mail, email	**#6 (Data collection):**<br>Face-to-face interview by home visits was conducted.
12. Sample size	How many participants were in the study?	**#8 (Results):**<br>35 caregivers of deceased newborn infants participated.
13. Non-participation	How many people refused to participate or dropped out? Reasons?	**#9 (Results):**<br>None of the caregivers refused or dropped out from the study.
Setting		
14. Setting of data collection	Where was the data collected? e.g. home, clinic, workplace	**#6 (Data collection):**<br>Data collection was conducted at each participant's home.
15. Presence of non-participants	Was anyone else present besides the participants and researchers?	It was not reported.

16. Description of sample	What are the important characteristics of the sample? e.g. demographic data, date	**#5 (Study sites):**<br>Detail information was provided in the **Supporting Information S1 Table**.
Data collection		
17. Interview guide	Were questions, prompts, guides provided by the authors? Was it pilot tested?	**#6 (Data collection):**<br>A validated instrument ('International Standard Verbal Autopsy Questionnaires Death of a Child Age Under 4 Weeks'), which was translated into Cambodian language, was used.
18. Repeat interviews	Were repeat interviews carried out? If yes, how many?	Not reported in the manuscript:<br>The interview session was once for each participant.
19. Audio/visual recording	Did the research use audio or visual recording to collect the data?	**#6 (Data collection):**<br>No audio/visual recordings were used for the interview.
20. Field notes	Were field notes made during and/or after the interview or focus group?	Not reported in the manuscript:<br>International Standard Verbal Autopsy Questionnaires Death of a Child Age Under 4 Weeks was used for making notes during the interview. All information from the participants was recorded in the questionnaire form.
21. Duration	What was the duration of the interviews or focus group?	Not reported in the manuscript:<br>Approximate duration of the interviews were between 20 and 30 minutes.
22. Data saturation	Was data saturation discussed?	Not reported in the manuscript:<br>Data saturation was not discussed because we intended to involve all the neonatal deaths in the selected villages.
23. Transcripts returned	Were transcripts returned to participants for comment and/or correction?	Not reported in the manuscript:<br>No, it was not returned.
**Domain 3: analysis and findings**		
Data analysis		
24. Number of data coders	How many data coders coded the data?	Not reported in the manuscript:<br>Three (AS, MM, and AI) were involved in the data extraction process.
25. Description of the coding tree	Did authors provide a description of the coding tree?	Not reported in the manuscript:<br>The coding tree was not provided.

26. Derivation of themes	Were themes identified in advance or derived from the data?	**#7-8 (Data analysis):**<br>The themes were derived from the data.
27. Software	What software, if applicable, was used to manage the data?	**#6-7 (Data analysis):**<br>No specific software was used for qualitative data analysis. Stata software was used for quantitative data analysis.
28. Participant checking	Did participants provide feedback on the findings?	Not reported in the manuscript:<br>No, they did not.
Reporting		
29. Quotations presented	Were participant quotations presented to illustrate the themes/findings? Was each quotation identified? e.g. participant number	**#11-13 (Results):**<br>Yes, they were. Several selected quotations were shown in each theme.
30. Data and findings consistent	Was there consistency between the data presented and the findings?	**#8-17 (Results and Discussion):**<br>Yes. There was no inconsistent information.
31. Clarity of major themes	Were major themes clearly presented in the findings?	**#11-13 (Results):**<br>Yes, they were. We identified three major themes.
32. Clarity of minor themes	Is there a description of diverse cases or discussion of minor themes?	Not reported in the manuscript:<br>No, because we have not identified minor theme.

Developed from:

Tong A, Sainsbury P, Craig J. Consolidated criteria for reporting qualitative research (COREQ): a 32-item checklist for interviews and focus groups. *International Journal for Quality in Health Care*. 2007. Volume 19, Number 6: pp. 349 – 357

Once you have completed this checklist, please save a copy and upload it as part of your submission. When requested to do so as part of the upload process, please select the file type: *Checklist*. You will NOT be able to proceed with submission unless the checklist has been uploaded. Please DO NOT include this checklist as part of the main manuscript document. It must be uploaded as a separate file.
