# Supplementary material for: "Why did our baby die soon after birth?"—Lessons on neonatal death in rural Cambodia from the perspective of caregivers
Source: PLoS One. 2021 Jun 7;16(6):e0252663. doi: 10.1371/journal.pone.0252663 (PMC8183999; doi:10.1371/journal.pone.0252663)
Supplement: S1 Table — (PDF) [file pone.0252663.s003.pdf]

S1 Table. Summary of the characteristic on demography and health service in Kampong Cham, Svay Rieng provinces, and Cambodia.

	Kampong Cham	Svay Rieng	Cambodia
Total population (2019) [1]	895,763	524,554	15,288,489
Population density (km^2) (2019) [1]	197	177	86
Sex ratio (Number of males per 100 females) (2019) [1]	91.7	90.7	94.3
Birth registration (%) (2014) [2]			
percentage who had a birth certificate	71.5	84.7	63.9
percentage who did not have birth certificate (but registered)	4.0	2.8	9.4
percentage registered	75.5	87.5	73.3
Antenatal care coverage at least one time (%) (2014) [2]	96.9	98.1	95.3
Doctor	17.6	0.5	5.7
Nurse	0.3	0.0	1.3
Midwife	79.0	97.6	88.3
Traditional birth attendants	0.0	0.0	0.1
Others	0.0	0.0	0.0
Birth attendance (%) (2014) [2]	91.5	94.3	89.0
Doctor	28.7	12.2	15.3
Nurse	1.4	0.0	3.2
Midwife	61.4	82.2	70.5
Traditional birth attendance	8.5	5.0	10.7
Village health volunteer	0.0	0.4	0.1
Others	0.0	0.2	0.0
Place of delivery (%) (2014) [2]			
Percentage delivered in a health facility	84.5	82.4	83.2
Health facility public	61.0	74.3	68.9
Health facility private	23.5	8.2	14.3
Home	15.2	17.2	16.6
Others	0.4	0.4	0.2
EmONC facilities (2015) [3]			
Number of BEmONC facilities (per 500,000 population)	N/A	N/A	1.04
Number of CEmONC facilities (per 500,000 population)	N/A	N/A	1.31
Number of BEmONC facilities			
Referral hospital	6	1	52
Health center	4	6	84
Number of CEmONC			
Referral hospital	1	2	44
Number of health care workers (per 10,000 population) (2014) [4]			
Medical doctor	N/A	N/A	1.93
Nursing and midwifery	N/A	N/A	9.62

References:

1. National Institute of Statistics, Ministry of Planning. General Population Census of the Kingdom of Cambodia 2019.
2. National Institute of Statistics, Directorate General for Health, and ICF International. Cambodia Demographic and Health Survey 2014. Phnom Penh and Rockville: National Institute of Statistics, Directorate General for Health, and ICF International; 2015.
3. Ministry of Health, Cambodia. EMEMERGENCY OBSTETRIC & NEWBORN CARE (EmONC) IMPROVEMENT PLAN 2016-2020. Phnom Penh: Ministry of Health;2016.
4. The World Bank, DataBank, World Development Indicators. Available online: https://databank.worldbank.org/reports.aspx?source=2&country=KHM# (Accessed on 26 Dec 2020)
