# Supplementary material for: "Why did our baby die soon after birth?"—Lessons on neonatal death in rural Cambodia from the perspective of caregivers
Source: PLoS One. 2021 Jun 7;16(6):e0252663. doi: 10.1371/journal.pone.0252663 (PMC8183999; doi:10.1371/journal.pone.0252663)
Supplement: S2 Table — (PDF) [file pone.0252663.s004.pdf]

S2 Table. Summary of the process from birth to death and suspected causes in 35 neonatal deaths cases in Kampong Cham and Svay Rieng provinces, between 2015 and 2016.

Case	Birth weight [kg]	Gestational month	Place of birth	Age of death [days]	Place of death	Symptoms / Signs	Diagnosis in hospital	Suspected causes of the death	Process from live birth to neonatal death
1	***1.8***	***7***	***Public referral hospital***	***2***	***Public referral hospital***	– ***Did not cry after birth*** – ***Respiration was weak***	***N/A***	***Prematurity***	***Preterm birth, recognized as dead by SBA, but found alive by the grandmother, delay of essential resuscitation***
2	1.2	7	Health center	2	Specialized children's hospital	– Difficulty in breathing – Vomiting	Esophageal atresia	Congenital disorder	Preterm birth, referred to a specialized hospital, diagnosed as esophageal atresia (congenital disorder)
3	2.7	9	Health center	25	Specialized children's hospital	– Fever – Diarrhea and bloody stool – Convulsion	Intra-cranial hemorrhage	Infection (sepsis)	Visited a health center because of convulsion, referred to a specialized hospital, diagnosed as intra-cranial hemorrhage
4	***2.0***	***7***	***Home***	***0***	***Home***	– ***Cold to the touch***	***N/A***	***Prematurity***	***Sudden preterm birth without SBA, became hypothermia***
5	2.0	7	Public referral hospital	1	Public referral hospital	– Pale skin color at birth	N/A	Prematurity	Preterm birth (the detail was unknown)
6	3.0	8	Home	0	Home	– Umbilical cord around the neck of baby – Mother fell down into a hole twice	N/A	Unknown	Born without SBA (the detail was unknown)
7	2.3	8	Home	0	Home	– Skin color changed to black	N/A	Unknown	Preterm birth with SBA (the detail was unknown)
8	1.4	7	Private clinic	1	Private clinic	– Fast breathing – Difficulty in breathing	N/A	Prematurity	Preterm birth, respiratory disorder (the detail was unknown)
9	***1.9***	***8***	***Health center***	***2***	***Home***	– ***Difficulty in breathing***	***N/A***	***Prematurity***	***Preterm birth, respiratory disorder, could not be put into an incubator***
10	3.0	9	Health center	0	On the way	– Difficulty in breathing – Chest indrawing – Grunting – Flaring of the nostrils	Anhydramnios	Asphyxia	Born at a health center, respiratory disorder, received oxygen and resuscitation, died on the way to home
11	2.7	9	Public referral hospital	5	Specialized children's hospital	– Fever – Vomiting – Difficulty in breathing	Esophageal atresia	Congenital disorder	Diagnosed as esophageal atresia (congenital disorder), referred to a specialized hospital, operated twice
12	2.9	9	Health center	4	Home	– Hemorrhage from nose and mouth	N/A	Unknown	Born at a health center and died at home due to hemorrhage (the detail was unknown)

13	1.5	7	Public referral hospital	28	Home	– Fever<br>– Cough<br>– Difficulty in breathing<br>– Chest indrawing<br>– Grunting<br>– Flaring of the nostrils	N/A	Prematurity	Preterm and twin birth, had been cared in an incubator, died after one day out of incubator
14	1.8	8	Home	14	Public referral hospital	– Difficulty in breathing<br>– Chest indrawing<br>– Grunting	N/A	Prematurity	Preterm birth with SBA, respiratory disorder, referred to a public hospital
15	3.0	9	Health center	27	Specialized children's hospital	– Fever<br>– Cough<br>– Difficulty in breathing<br>– Vomiting	Hepatic cyst	Congenital disorder	Vomited at home, referred to a specialized hospital, diagnosed as hepatic cyst (congenital disorder), died after a few hours of operation
16	4.0	9	Private clinic	0	Private clinic	– Difficulty in breathing<br>– Grunting	N/A	Asphyxia	Respiratory disorder (the detail was unknown)
17	3.1	9	Private clinic	2	Public referral hospital	– Difficulty in breathing<br>– Flaring of the nostrils	N/A	Asphyxia	Prolonged labor, respiratory disorder, referred to a public hospital
18	2.0	7	Health center	0	Health center	– Twin pregnancy<br>– Pale skin color at birth	N/A	Prematurity	Preterm and twin birth, without referral
19	***2.0***	***7***	***Health center***	***0***	***Home***	– ***Twin pregnancy***<br>– ***Pale skin color at birth***	***N/A***	***Prematurity***	***Preterm and twin birth, died at home without referral***
20	3.5	9	Health center	2	Home	– Fever after vaccination<br>– Skin rash and eye redness<br>– Vomiting<br>– Hemorrhage	N/A	Adverse effect of vaccination	Fever up after one hour of vaccination, given paracetamol by her mother, had allergic symptoms and died at home
21	3.1	9	Public referral hospital	17	Public referral hospital	– Fever<br>– Difficulty in breathing<br>– Chest indrawing<br>– Grunting<br>– Flaring of the nostrils	Pneumonia	Infection (pneumonia)	Fever up for one day, visited a public hospital, diagnosed as pneumonia
22	***4.3***	***9***	***Home***	***1***	***Home***	– ***Umbilical cord was cut after a few hours of birth***	***N/A***	***Unknown***	***Born without SBA, delay of essential immediate newborn care such as cutting umbilical cord***
23	3.0	7	Private clinic	0	Private clinic	– malformation	Anencephalus	Congenital disorder	Preterm birth, fatal congenital disorder (anenchephalus)
24	2.9	9	Health center	20	Home	– Vomiting<br>– Hemorrhage from anus	N/A	Infection (intestinal)	Started vomiting, could not suck, treated at a health center
25	***1.2***	***9***	***Public referral hospital***	***0***	***Public referral hospital***	– ***Difficulty in breathing***<br>– ***Flaring of the nostrils***	***N/A***	***Prematurity***	***Preterm birth, respiratory disorder (the detail was unknown)***
26	1.3	8	Health center	17	Public referral hospital	– Difficulty in breathing	Congenital heart disease	Congenital disorder	Preterm birth, respiratory disorder, referred to a public hospital, diagnosed as congenital heart disease

27	***1.3***	***8***	***Health center***	***0***	***Home***	– ***Difficulty in breathing***<br>– ***Flaring of the nostrils***	***N/A***	***Prematurity***	***Preterm birth, respiratory disorder, without referral***
28	1.4	7	Health center	0	Home	– Difficulty in breathing	N/A	Prematurity	Preterm birth, respiratory disorder (the detail was unknown)
29	2.7	7	Public referral hospital	15	Specialized children's hospital	– Difficulty in breathing	Lung disease	Infection (pneumonia)	Preterm birth, respiratory disorder, diagnosed as pneumonia, referred to a specialized hospital
30	2.5	8	Midwife's home	3	Home	– Blue skin color	N/A	Unknown	Preterm birth (the detail was unknown)
31	1.3	7	Public referral hospital	15	Home	– Difficulty in breathing<br>– Grunting<br>– Flaring of the nostrils	N/A	Prematurity	Preterm birth, respiratory disorder, treated at a public hospital (the detail was unknown)
32	1.5	8	Public referral hospital	6	On the way	– Vomiting<br>– Abdominal distention	N/A	Prematurity	Preterm birth, treated at a public hospital and died on the way (the detail was unknown)
33	2.0	8	Public referral hospital	0	Public referral hospital	– Breech presentation at birth<br>– Parity = 0	N/A	Asphyxia	Preterm birth, treated at a public hospital (the detail was unknown)
34	2.7	9	Home	0	Home	– Malformation	N/A	Congenital disorder	Born with malformation at home and died (the detail was unknown)
35	2.7	9	Health center	3	Public referral hospital	– Difficulty in breathing<br>– Chest indrawing<br>– Grunting	N/A	Respiratory distress (the detail was unknown)	Born at health center, respiratory distress, referred to a public hospital (the detail was unknown)

Notes: Cases written by Bold Italic characters are presented as quotations in the main text.
