# Supplementary material for: "Why did our baby die soon after birth?"—Lessons on neonatal death in rural Cambodia from the perspective of caregivers
Source: PLoS One. 2021 Jun 7;16(6):e0252663. doi: 10.1371/journal.pone.0252663 (PMC8183999; doi:10.1371/journal.pone.0252663)
Supplement: Attachment — Submitted filename: Response to Reviewers.docx [file pone.0252663.s005.docx]

**PONE-D-20-32314
"Why did our baby die soon after birth?" – lessons on neonatal death in rural Cambodia from the perspective of caregivers.**

**Additional Editor Comments (if provided):**
This is an interesting article in the field of maternal and child health in a lower-middle income country. Its contents will be improved by responding to the specific comments from two reviewers. The following points should be also considered to improve its quality.

| **Comment** | **Point by point responses** | **Changed part in the manuscript (new Line)** |
| --- | --- | --- |
| L20: The abstract should be revised after improving the main text. | The abstract was revised according to the revised main text. | **Abstract** |
| L24: This study aimed to…(the same for the main text) | I changed the sentence in the main text to the same style as **Abstract**. | (L4-6 and L65-66) **[Introduction]** with the aim of…  → (L4-6 and L65-66) The study aimed to … |
| L73: The objective statement is not strong enough. “Drawing lessons” are too general. It should be more specific. | I changed the objective statement. | (L4-6 and L65-66) This study aimed to identify the barriers to reduce preventable neonatal deaths in rural Cambodia. |
| L79: This study seems to be conducted by using a mixed methods research design, but it was not considered as such. If not, please explain it. The authors’ may read several articles about the mixed methods research, for example, by Creswell and reconstruct the methods section. For qualitative research part, it is better to attach COREQ check list. It will strengthen the credibility of the narrative records. It is also better to write how “validity threats” were overcome. (See Maxwell JA. Qualitative research design: an interactive approach. 3rd ed. SAGE, 2013.) The method of data analysis is not clear, either. | Thank you for pointing this important issue. .We concluded that this is a qualitative case study research referring the suggested document (Creswell, 2013). We used quantitative data only for neonatal mortality estimation. No other quantitative information from verbal autopsy was analysed. We attach the COREQ check list as a part of supporting information. | At the beginning of **Method** section (L68-69), I add the following sentence: ‘This study adopted the method of qualitative case study research using narrative data from the verbal autopsy’ |
| L 139: For each %, % is not necessary. Just 34 is enough. The same for Table 2. | I deleted all ‘%’. | **Table 1** & **Table 2** |
| L196: In many paragraphs, the first sentences started with general statement, and then it was supported by the authors’ findings. This style should be changed, not from general to specific, but from specific to general. Please show the finding of this study first and then discuss it using references and other findings of this study. One paragraph is too long to explain each topic sentence. | - I rewrote the first sentence more specific as the topic sentence as much as possible. - I made each paragraph shorter to explain each topic sentence clearer than before. | - |
| L 198, 199: This sentence is not necessary. Please make a summary of major findings. “Reveal” is a very strong verb, which should be avoided in this study design. | The verb was modified as identified; and the sentence were modified. | (L207-8) Our findings identified several challenges for implementing interventions to save newborn lives, such as skilled birth attendance at delivery, improvement of quality of care, and enhancement of referral systems . |
| L203: It looks like “causality” was proven about the unavailability of SBAs in this study. | Sentences were modified. | (L210-1) Potential causality of neonatal deaths was indicated in this study, which was the unavailability of SBAs due to too rapid progress of delivery, or inaccessibility to health facility due to heavy weather. |
| L257: Please add one topic sentence before starting the paragraph. | I added one topic sentence at the beginning of the paragraph. | (L269-70) Major limitation of this study was in the retrospective nature of verbal autopsy, especially in recall bias among the respondents.. |
| L310: The Royal Government… | I modified the reference. | Reference #9  →　The Royal government of Cambodia. National Strategic Plan of Identification 2017-2026. Phnom Penh: The Royal government of Cambodia; 2016. |
| Fig 1: It is not clear. | To show the results clearer, we created a table (Table 1) instead of previous Figure 1. | Table1. Collected information on births and neonatal deaths in year 2015 and 2016,  Kampong Cham and Svay Rieng provinces, Cambodia |

Reviewer #1: This study analyzed the results of verbal autopsy for neonatal mortality cases in two rural provinces in Cambodia. It highlights important barriers that might affect neonatal death in a country that is often regarded as a success case for improving maternal, newborn, and child health. I believe that the insights presented in the manuscript should be widely shared. I hope the points listed below would contribute to improving the manuscript, if relevant.

| **Comment** | **Response** | **Changed part in the manuscript (new Line)** |
| --- | --- | --- |
| 1. The authors stated that “the reduction of neonatal deaths has stagnated.” And they presented data as “From 1990 to 2018 the under-five mortality rate per 1,000 live births dropped by 59%, from 93 to 39, while neonatal mortality was reduced by 52%, from 37 to 18 [1].” (Line 2-) This shows that the percentage of reduction is 59% for under-five mortality and 52% for neonatal mortality. It sounds a bit too strong to state that “the reduction of neonatal deaths has stagnated” compared to child mortality. | I changed the first sentence and deleted the word:’ stagnated’. | (L24-5) As the total child mortality has deceased globally in the past several decades, the percentage of neonatal death among them has been relatively increasing [1]. |
| 2. In the **Introduction** section, the authors can explain how verbal autopsy has been implemented in resource-limited settings, by referring to previous studies that tackled its possible methodological issues. These issues may include how to improve the identification of possible causes of deaths, particularly when death certificates are not available and when the recall period is longer than the recommended period? The authors may notice that WHO recommended that the recall period does not exceed one year of death (for example, <https://www.who.int/healthinfo/statistics/WHO_VA_2012_RC1_Instrument.pdf?ua=1>). The authors may relate these issues to be mentioned in the **Background** section to the **Methods** section (how the data collection minimizes potential errors) and the **Discussion** section (strengths and limitations of this study). | 2.  1) An explanation on the verbal autopsy is added.  2) Training process of research assistants to minimize potential errors for the verbal autopsy was added.  3) The causes of early neonatal death within seven days after birth, which covers more than three-forth among all neonatal mortality linked each other, such as ‘infection with prematurity’. It is, therefore, difficult to detect the cause of death by verbal autopsy only. There are several scientific methods to identify one reason for death by more than two specialists (ex: <http://www.byass.uk/interva/>  <https://www.tandfonline.com/doi/full/10.3402/gha.v5i0.19281> ) but not feasible in resource-limited settings. So, this study aimed not to clarify causes of neonatal death themselves but to identify the barriers to be solved from the viewpoints of health system to reduce preventable neonatal deaths.’  4) In **Data collection and analysis** of **Method** section, I add the explanation why we set two years as the recall period. | 1) (L61-3) Because of its availability, verbal autopsy approach with written interview reports has been applied in many resource-limited settings [12] 2) (L80-3) All members had experience in conducting interviews in the latest Cambodia Demographic Health Survey in 2014. The surveyors received a three-days training, which aims to reconfirm and consolidate proper skills of interview with the standard questionnaires as well as to ensure the process of data collection. 3) as it is explained in the left cell. 4) (L91-3) We set the recall period longer than WHO recommendation (two years versus one year) because the estimated mortality cases per one year were limited according to the national data.’ |
| 3. The authors may explain more about the “Study sites” subsection (Line 80-). Were there any activities (other than verbal autopsy) in the JICA project, which could potentially reduce neonatal mortality? It is unclear if the verbal autopsy in this study could be interpreted as the one without project intervention or not. | 3. This study was conducted as the initial situation analysis at the beginning of JICA project as I mentioned in Introduction (L66-68). So, the results of verbal autopsy were not affected by the project intervention. |  |
| 4. Related to the point above, were there any ongoing or previous external assistance (by JICA and other organizations) in the study sites related to neonatal health? This information may be important to interpret neonatal mortality rate estimates and the cause of deaths in this study. | 4. In **Introduction**, I add the sentence to explain the previous external assistance. | (L54-7) In Kampong Cham, World Health Organization supported the coaching session on immediate newborn care from the year 2013 to 2015 while there were no previous external assistances on newborn health in Svay Rieng provinces before this study. |
| 5. Related to the point above, the “Study sites” subsection may need more about the level of maternal, newborn, and child health services in the study sites, in addition to neonatal mortality rates. Such information may include the followings: the coverage of facility-based delivery, delivery assisted by skilled birth attendants, and the mean number of antenatal care received. In addition, the authors may need to explain the hierarchical structure of health facilities, the availability of midwives and delivery assistance in different tiers of health facilities, the availability of transportation means owned by a health facility (such as ambulance), and the activities of village-level health volunteers, if any, in the study site. Such information may help readers to interpret the causes of deaths presented in this study and understand the Discussion section. | 5. We added the appendix as the **Supporting Information Table 2** to provide relevant information, including the demography, birth registration rate, the proportion of receiving ANC, the proportion of SBA delivery, the place of delivery, structure of health facilities and the number of health workers. I added one sentence at the end of **Study sites in Methods**. | The detailed health information is shown in **Supporting Information Table 2.** |
| 6. How was the training of survey team members conducted? Is there any supervision or auditing of survey team members to improve the quality of the cause of death identification? They may be related to how this study minimized biases in the cause of death identification. | 6. I added the sentence about the training for survey team members in **Methods** section. | (L80-3) All members had experience in conducting interviews in the latest Cambodia Demographic Health Survey in 2014. The surveyors received a three-days training, which aims to reconfirm and consolidate proper skills of interview with the standard questionnaires as well as to ensure the process of data collection. |
| 7. How were neonatal death cases distinguished from stillbirth cases? The questionnaire used in this study may be able to distinguish between them, if it is properly used. The authors could explain how to minimize potential errors. | 7. All neonatal death cases were distinguished from stillbirth by continuous/plural questions from birth to death narratively. |  |
| 8. Reference number 12 ('International Standard Verbal Autopsy Questionnaires Death of a Child Age Under 4 Weeks') is the 2007 version. Are there reasons why the authors did not use the 2012 version or later? | 8. We used the 2007 version because there had been already Khmer version for 2007. Before the survey, we had checked there were not crucial difference between the 2007 and 2012 version in English. |  |
| 9. In Lines 101-, the authors explained how the suspected causes of deaths were documented. In the data collection, how were the data items in the Supplementary Table (such as the birth weight, gestational months, and diagnosis in hospitals) identified? Did survey team members refer to any documents that interviewees held or a health facility possessed? What if these documents were unavailable? The authors could explain more about data collection process and validation. | 9. I added the explanation on the documentation of death cases. | (L99-102) The surveyors referred the official documents such as maternal health record book or immunization card, if the interviewees had kept it. In cases with any records, information which were referred to suspect the causes of death relied on memories of caregivers only. |
| 10. In Lines 118-, the authors explained that “neonatal mortality rates were also similar in the two provinces at approximately 8 per 1,000 live births.” Did this estimate reflect the complex survey design the authors used (two-stage cluster sampling)? The authors concluded that the estimated neonatal mortality rates were lower than the provincial estimates, according to DHS. However, the authors may be able to estimate the confidence interval and interpret if the estimate is different from the DHS estimate even after considering the confidence interval. | 10. Thank you for pointing out our fault. We have recalculated neonatal mortality rate with its confidence interval considering sample weight. | The results is shown in Table 1 in the revised manuscript. |
| 11. Related to the point above, the authors could note that DHS estimates of neonatal mortality were possibly influenced by the undercount of mortality cases and thus corrected by multiple methods (for example, <https://www.un.org/en/development/desa/population/publications/pdf/technical/TP2011-2_MortEstMajorSampSurv.pdf>). That is, neonatal mortality rate based on DHS may be corrected its undercounts. Therefore, the authors’ neonatal counts might not necessarily be worse than counts done by DHS. The authors may be able to justify their methods by pointing out that such undercounts might be inevitable. | 11. Thank you for suggesting the reference as well as the potential reason for the difference between our findings and Cambodia DHS (CDHS) 2014 results. We have added several speculations on the differences in our discussion part. Since we have no means to adjust the neonatal mortality rates with the small samples, and our principal aim is not in the estimation, the aim to show the comparison with the CDHS data is to present that there was possible underestimation in our survey. | (L275-81) Potential reasons for underestimation of neonatal mortality rates are added in the discussion part. |
| 12. The authors analyzed narratives by focusing on services related to childbirth (such as availability of SBAs, knowledge and skills related to neonatal deaths, and referral) (Lines 153-). I totally agree that these factors are important to reduce neonatal deaths. In addition, were there issues related to services in the pregnancy stage identified, such as early detection and management of maternal complications and advice related to birth preparedness? | 12. I agree with the reviewer’s comment. I added one sentence in **Discussion** section. | (L236-8) In addition, services during antenatal care for pregnancy, such as early detection and management of maternal complications and advice related to birth preparedness affect neonatal outcome [23]. |
| 13. The authors proposed the training of lay village workers dispatched in rural communities to improve access to SBAs (Line 206-). Similar efforts might have already been started in, at least, some part of Cambodia (for example, <https://resourcecentre.savethechildren.net/node/16699/pdf/Learning%20package%202%20-%20Role%20of%20volunteers%20FINAL_low%20res%20for%20WEB.pdf>;<http://unicefcambodia.blogspot.com/2015/04/volunteers-crucial-to-health-delivery.html>). The authors may be able to explain how it could work in the study site, by referring to past efforts in Cambodia. | 13. Thank you for your useful reference. Based on this information, I added one sentence in **Discussion** section. | (L223-5) The past experience in Stung Treng province in Cambodia tells us the key to success of community involvement depends on both leadership and teamwork such as village health support groups approved by the ministry of interior [20]. |
| 14. The authors may be able to document their lesson learnt for those who conduct verbal autopsy, by focusing on how verbal autopsy can be improved at resource-limited settings. | 14. As one limitation, I added one sentence and started the new paragraph after that. | (L271-2) Such blurred memories may cause the  surveyors’ assumptions which were recorded as the causes of deaths. We need to emphasize the interviewers should dictate all the narratives accurately without modification in their trainings. |
| 15. I believe the data has been made available in Supplementary Table, on the contrary to the data availability statement (“some restriction may apply”). | 15. Thank you for your pointing out.  Original data of narrative parts included plural individual privacy and sensitive information such as names of mothers or health centers. These data cannot be disclosed according to our research protocol which was approved by ethical committees both in Cambodia and Japan. |  |

Reviewer #2:

| **Comment** | **Response** | **Changed part in the manuscript (new Line)** |
| --- | --- | --- |
| [Abstract] 1. Please add the total number of births identified in the study sites. | 1. We added the total number of births identified. | （L12-3） Among a total of 4,142 children born in 2015 and 2016, 35 neonatal deaths were identified. |
| [Introduction] 2. Please mention the birth registration rate in Cambodia. (Line 60) | 2. - We added the birth registration rate in Cambodia.  - We also provided the characteristic of the health service in **Supplementary Information Table 2**. | - (L46-8) The national birth registration rate in Cambodia was estimated around 73% of children under five years old in 2014 while National Strategic Plan of Identification is targeting 90% of birth registration in a given year by 2024 [9]. - **Supplementary Information Table 2** |
| 3. Please provide common reasons for underreporting of death in Cambodia. | 3. Based on the Cambodian Civil Code and Sub-decree No. 103, parents need to report a birth within 30 days. However, births are not often registered timely manner. Sometimes parents lack in knowledge and they do not report until the registrations are required (e.g. school enrolment). Even though parents are aware of the duty, it takes several weeks to register. Also, most of neonatal deaths are occurred at home and most of them do not have a death certificate. As a result, many neonatal births and deaths are not registered.  [References]   - United Nations Development Program   Human Development Report. Why birth and death. registration really are “vital” statistics for development  [http://hdr.undp.org/en/content/why-birth-and-death-registration-really-are-“vital”-statistics-development](http://hdr.undp.org/en/content/why-birth-and-death-registration-really-are-)   - AbouZahr C, de Savigny D, Mikkelsen L, Setel PW, Lozano R, Nichols E, Notzon F, Lopez AD. Civil registration and vital statistics: progress in the data revolution for counting and accountability. Lancet. 2015 Oct 3;386(10001):1373-1385. |  |
| 4. Please provide general information on maternal newborn and child health care program in Cambodia, including healthcare system, types of health care providers, and health insurance for health care services. | 4. General information related to maternal and child health, including birth registration rate, ANC attendance and place of delivery are provided in **Supprting Information Table 1**. | **Supporting Information Table 1** |
| 5. How many days do mothers and babies stay at facility after giving birth? | 5. The government of Cambodia has adopted the recommendations on postpartum care from WHO.  If birth is at a health facility, mothers and newborns should receive postnatal care there for at least 24 hours after birth. If birth is at home, the first postnatal contact should be as early as possible within 24 hours of birth. At least three additional postnatal contacts are recommended for all mothers and newborns, on day 3 (48–72 hours), between days 7–14 after birth, and six weeks after birth.  [Reference]  Ministry of Health, Cambodia. The Safe Motherhood Clinical Management National Protocol, Health Centers. Phnom Penh: Ministry of Health; 2016. |  |
| 6. Please replace some information about study design and setting (Lines 63-72) to the Method section. | 6. The information has been replaced in the method section. |  |
| [Methods] 7. In the study site section, please provide the population of the two provinces. | 7. We provided the population size in the new **Table 1** and **Supporting Information Table 1**. |  |
| 8. Please describe more clearly about the second stage of sampling because I wondered if authors randomly selected villages per a commune after determining the number of villages to be selected per commune in advance, or randomly selected 80 villages from 8 communes in Kampong Cham and 40 villages from 4 communes in Svay Rieng regardless of commune size. (Lines 88-94) | 8. Thank you for your perceptive comment on this matter. I changed the explanation on sampling method. | (L84-9) The target villages were selected by following procedure. First, we have randomly selected eight and four communes in Kampong Cham and Svay Rieng provinces, respectively. Then, 80 and 40 villages were randomly selected from the communes for respective province. The number of target villages per province was weighted according to the number of inhabitants in the two provinces, as well as the limitation of budget and duration of the survey. |
| 9. Please provide the reference for the analysis of verbal autopsy. (Line 104) | 9. We followed the guidance from the WHO standard [Reference 13]. Another technical guide was also referred on how to translate without biases: <https://crvsgateway.info/file/11243/3231>. |  |
| 10. Please explain more detail about how the narrative data on the process from live birth to neonatal death was analyzed. (Lines 104-106) | 10. We added the key words to identify potential issues in health service. | (L11-3) Information of the illness and events (Question 401) and given treatment (Question 1102) were extracted using keywords such as ‘premature’, ‘weak’, ‘small’, ‘deliver at home’ |
| 11. Verbal autopsy potentially gives emotional distress to respondents. How did the study consider for it? | 11. Thank for your precious comment. We always paid attention to this matter. In the explanation leaflet as a part of research proposal, which was submitted to and approved by the ethical committee, we documented: ‘The participation in this interview is up to your (the caregivers’) will. You can always withdraw the interview if you want anytime.’ |  |
| [Results] 12. Please show the number of neonatal death cases first, followed by neonatal mortality rates. (Lines 118-121) | 12.We changed the order accordingly. |  |
| [Discussion] 13. Please discuss the results of neonatal mortality rates and suspected causes of death, although the authors have explained potential reasons for the lower neonatal mortality rate observed in the study compared to the national representative data. | Thank you very much for your precious comments. We only identified 35 death cases which of the size is too small to discuss the statistical difference to the national neonatal mortality rate. In addition, it is not easy to identify the single cause of neonatal death because neonatal death is normally the result of complicated chain of multiple events. In this study, we focused on finding the avoidable events which possibly lead to the neonatal death rather than identifying the single cause of death of each case. |  |
