# Supplementary material for: "Why did our baby die soon after birth?"—Lessons on neonatal death in rural Cambodia from the perspective of caregivers
Source: PLoS One. 2021 Jun 7;16(6):e0252663. doi: 10.1371/journal.pone.0252663 (PMC8183999; doi:10.1371/journal.pone.0252663)
Supplement: Attachment — Submitted filename: 0315 Response to Reviewers.docx [file pone.0252663.s006.docx]

**PONE-D-20-32314RI
"Why did our baby die soon after birth?" – lessons on neonatal death in rural Cambodia from the perspective of caregivers.**

**Additional Editor Comments (if provided):**
**Now the research design is clear. To improve the quality of the manuscript, please try to improve the following points.**

| **Comment** | **Point by point responses** | **Changed part in the manuscript (new Line)** |
| --- | --- | --- |
| 1. P2L1: In the abstract, please add subheadings like other PLoS One articles: Introduction, Methods, Results, and Conclusion. | 1. The abstract was revised with subheadings like other PLoS One articles. | **Abstract** |
| 2. P2L3: …neonatal deaths have not been well described… | 2. The sentence is changed accordingly. | **Abstract** P2L4: …neonatal deaths have not been well described. |
| 3. P2L5: aimed to…(In this type of research, the objective statement should be written in the past tense.) | 3. The tense is now changed. | **Abstract**  P2L5: aimed to… |
| 4. P2L6: This study adopted a qualitative case study design using… | 4. The sentence is changed accordingly. | **Abstract**  P2L8: This study adopted a qualitative case study design using… |
| 5. P2L12: Please add a data analysis method. | 5. A data analysis method is now added. | **Abstract**  P2L12-14: Information on the process from birth to death was extracted with open coding, categorized, and summarized into several groups which represent potential problems in health services. |
| 6. P2L14: Narrative data showed that the following three (themes? factors?) should be improved……mortality. They were insufficient availability…….., and barriers in referral. | 6. The sentence is changed, with shorter expression because of word counts. | **Abstract**  P2L18-20: Narrative data showed that three factors should be improved, 1) the unavailability of a health-care professional, 2) lack of knowledge and skill to manage major causes of neonatal deaths, and 3) barriers in the referral system. |
| 7. P2L17: …could be also effective to facilitate…..residents for reducing neonatal mortality… | 7. This sentence has been deleted in revision and replaced with another sentence. | P3L24-26: Appropriate solutions in each community should be sought according to the differing situations in each setting. Revisiting the principles of primary health care will enable us to overcome the issues. |
| 8. P4L65: This study aimed to… | 8. The tense is now changed. | P5L75: This study aimed to… |
| 9. P5L68: This study adopted a qualitative case study design using… | 9. The sentence is changed accordingly. | P5L79: This study adopted a qualitative case study design using… |
| 10. P5L78: This comment is optional. In this section two writing styles are used and mixed up: “We…” or using “passive tense” without using “we.” This affects readability and it is better to stick to one style. Please decide whether to keep as it is, or to make the style consistent. | 10. In revision the passive tense is chosen without using “we” and the sentence modified accordingly. | P6L90-91: …the lists of all villages were obtained from the two provincial health departments, and… |
| 11. P5L71: Prior to qualitative data collection, a survey was conducted in… | 11. To make the explanation on the survey procedure clearer, several sentences were moved in this section, and the relevant sentence changed accordingly. | P6L90: Prior to the survey… |
| 12. P5L75: …which were higher than the national average….live births in the same year (OK?) | 12. The sentence is now changed accordingly. | P6L86-87: …which were higher than the national average….live births in the same year |
| 13. P5L81: …, which aimed to reconfirm… | 13. The phrase is now changed. | P7L113: … to help reconfirm… |
| 14. P6L103: Data analysis method is not clearly stated in here. Thematic analysis, grounded theory, or any other method was used for data analysis? | 14. In revision the first paragraph of **Data analysis** is changed with additional explanation on how to treat the data with qualitative case study method. | P7L115-127:  **Data analysis** part in the **Method** section |
| 15. P12L194: There were… | 15. The tense is now changed. | P13L221: There were… |
| 16. P12L211: It is a risky to use the term “causality” even if “potential” is added. You may simply say, “The first challenge is the unavailability of SBAs due to….” | 16. The sentence is now modified accordingly. | P13L240-241: The first challenge is the unavailability of health-care　professionals due to… |
| 17. P12L213: This is a recommendation statement, which is usually not appropriate to do it in the middle of discussion. All the recommendations should come before or after the final conclusion. This sentence can be rewritten as follows, for example. “To realize delivery by an SBA, two approach has been taken. The first one is to improve it from the service supply side. In India, for example, lay village health workers were involved and trained in rural communities, where SBAs were not easily available (16,17).” | 17. This sentence is now revised accordingly. | P13L241-P14L245: There are two possible approaches to overcome these difficulties. One approach is to ensure the proximity of essential interventions in the case of emergency. In India, for example, lay health workers were involved and trained in rural communities, where SBAs were not easily available [17,18]. |
| 18. P13L218: …had reduced neonatal… (Please use “verbs” instead of “noun +of”. Overusing nouns makes a sentence unreadable.) | 18. In revision the sentence is changed using verbs instead of “noun +of”. | P14L247: …successfully reduced neonatal… |
| 19. P13L224: …emergency. In Stung Trend province in Cambodia, the key to successful community involvement depends on… | 19. After rechecking the reference and consideration, this sentence is now modified and moved it to the last of next paragraph. | P15L265-268: Successful experiences have been described in rural Cambodia where lay health workers played important roles in educating mothers in communities as well as accompanying women to health facilities [23]. |
| 20. P13L227: The second approach for improving delivery by SBAs is strengthening (or empowering?) the demand side. By this, community people can raise awareness about safe delivery. The neonatal… | 20. Thank you for your comment. I changed the sentence. | P14L255-256: Another possible approach is empowering community people by raising awareness on safe delivery. |
| 21. P14L242: It sounds like you are discussion ref. 24, and your finding is used as a supporting evidence for the ref 24. Although it is not supporting the message of ref. 24, the key message in this paragraph seems to be ref. 24 and not yours. This paragraph is too long and redundant. | 21. In revision this paragraph is shortened with the key sentence at the beginning (“The second challenge is how to ensure quality care.”) . | P15L269-283: The fourth paragraph in the **Discussion** section |
| 22. P15L261: Again, you are discussing ref. 21 and 30. Please show your finding first and discuss it, not someone’s. On L209, “enhancement of referral systems” is used as one of major findings, this term should appear in the first sentence, not in the last one. | 22. In revision the structure of this paragraph is modified with the key sentence at the beginning: “Enhancement of referral systems is the third challenge, and an unfinished agenda in resource-limited settings” at the beginning. | P15L284-297: The fifth paragraph in the **Discussion** section |
| 23. P16L287: Again you are discussing ref 34, 35, and 37, rather than discussing your unique findings. It sounds like you are repeating “Introduction” in here and because of this, this last paragraph is very weak to show the strength of this study. Despite several limitations mentioned in the manuscript, what you can say with confidence? | 23. In revision we have deleted this paragraph because the contents are not at the center of our point. The last paragraph is now added as the final conclusion. | P17L316-325: The last paragraph in the **Discussion** section |

Reviewer #1:

The authors added more explanations on the study site, the method of a verbal autopsy, data collection and analysis, interpretations, and potential biases. I have several comments on the revised part of the manuscript.

| **Comment** | **Response** | **Changed part in the manuscript (new Line)** |
| --- | --- | --- |
| 1. Regarding the added explanations on neonatal mortality estimates in the Methods section, the authors may need to explain the methods of estimating the confidence interval by clarifying a particular estimation method (for example, Rao-Scott Chi-square test) or particular Stata command. | 1. In the revised version a new second sentence of **Data analysis** is added with additional explanation on estimation method using Stata software with the *svyset* command to adjust for the sampling weights. | P7L128-P8134: **Data analysis** part in the **Method** section |
| 2. In contrast to the revised objective statement (…to identify the problems in health care service), the Data collection subsection in the Methods section did not specifically mention how such problems in health care service were collected (the authors explained how mortality cases were identified and how the process of pregnancy, birth, signs, and symptoms were identified, though). The authors may clarify in the Methods section regarding how 'International Standard Verbal Autopsy Questionnaires Death of a Child Age Under 4 Weeks' were used to identify problems in health care service. | 2. In the main part of 'International Standard Verbal Autopsy Questionnaires Death of a Child Age Under 4 Weeks', there are no direct questions about health care service for caregivers. So we extracted the process from births to deaths from the narrative parts (Question 401 and 1102) and analyzed them from the viewpoints of health care service. Based on this situation, I added some explanation in the **Data collection** and **Data analysis** parts of **Method** section. | P6L89-P8L134: **Data collection** and **Data analysis** parts in the **Method** section |
| 3. As the authors characterize this study as a qualitative study in the revised manuscript, they may want to explain the methods according to the standard guidelines, not simply added the checklist. Regarded “N/A” items in the added COREQ checklist, the authors may be able to clarify the following items in the main text and the checklist: #2-#4 (background of 40 survey team members), #13 (whether there were non-participations among identified cases), #19 (clarify if audio/visual recording was not used), #20 (“International Standard Verbal Autopsy Questionnaires Death of a Child Age Under 4 Weeks” were used, so can be clarified in the checklist), #21 (typical time duration for the interview), #23 (clarify no if not returned), and #24-25 (coding strategy). | 3. In revision the Supporting Information S2 Checklist (CPREQ checklist) is modified and information is included about the methods according to the standard guideline in the main text (**Data collection** part of the **Method** section). | 1) **Supporting Information S2 Checklist** (COREQ checklist): #2-#4, #13, #19, #20, #21, #23, and #24-25. 2) P6L89-P7L114:**Data collection** part in the **Method** section |
| 4. Related to the comment above, the authors may be able to clarify how to extract themes from the verbal autopsy record. The authors identified “unavailability of a SBA,” “lack of knowledge and skills to manage major causes of neonatal deaths,” and “Barriers in referral.” How did the authors finally achieve to confirm these themes? | 4. We basically referred the ‘three-delay model’ for categorizing all narrative data although the content of each case was mixed. In revision we have added explanation on ‘three-delay model’ in the **Data analysis** part of the **Method** section. | P7L115-127: **Data analysis** part in the **Method** section |

Reviewer #2:

Thank you for addressing all my comments. I have a few more comments as follows.

| **Comment** | **Response** | **Changed part in the manuscript (new Line)** |
| --- | --- | --- |
| 1.I suggest to add the authors' responses in the main text: #5 the national recommendation on postpartum care, and #11 ethical considerations for respondents during verbal autopsy. | 1. Thank you for your comment.  1) In revision we have added information on #11 ethical considerations.  2) Regarding #5, now is the transaction period to increase the timings of postpartum care, so we did not mention it in the main text to avoid reader confusion. | P8L140-141: The participation was voluntary, and the participates could declare withdrawal from the study even after provision of the content. |
| 2. Thank you for creating S2 Table. This is very informative. I suggest the authors to add simple descriptions or key words regarding the process from live birth to neonatal death for each case. | 2. In revision we have added a new column for simple description at the right side on the process from live birth to neonatal death for each case. | **Supporting Information S3 (former S2) Table** |
| 3. I agree that the discussion in the last paragraph is important. However, I do not really see it the conclusion of the manuscript. Please provide a clearer conclusion. | 3. After consideration, we have deleted the former last paragraph and rewrote it as the final conclusion. | P17L316-325: The last paragraph in the **Discussion** section |
