# Supplementary material for: "Why did our baby die soon after birth?"—Lessons on neonatal death in rural Cambodia from the perspective of caregivers
Source: PLoS One. 2021 Jun 7;16(6):e0252663. doi: 10.1371/journal.pone.0252663 (PMC8183999; doi:10.1371/journal.pone.0252663)
Supplement: Attachment — Submitted filename: 0425 Response to Reviewers.docx [file pone.0252663.s007.docx]

**PONE-D-20-32314RI
"Why did our baby die soon after birth?" – lessons on neonatal death in rural Cambodia from the perspective of caregivers.**

**Additional Editor Comments:**

The revised manuscript was greatly improved. In addition to two reviewers' additional minor comments, please try to improve the following points.

| **Comment** | **Point by point responses** | **Changed part in the manuscript (new Line)** |
| --- | --- | --- |
| 1. Abstract: The verb 'reveal' is often used in more rigor study design articles. It is better to use 'show' or other weaker verbs. As two reviewers are suggesting to modify the conclusion of the main text, the conclusion of the abstract also may be slightly changed. | 1. The **Abstract** is revised accordingly. | **Abstract** |
| 2. P15 L273: "unfortunately" can be cut. | 2. The words: ‘and unfortunately,’ are deleted accordingly. |  |

**Reviewer #1:** 
Thank you very much for considering the comments made by reviewers provided in the previous round or the peer-review process. I have minor comments as follows:

| **Comment** | **Response** | **Changed part in the manuscript (new Line)** |
| --- | --- | --- |
| 1. In Lines 130-, the authors stated that “We considered that each village is a cluster and assumed that similar crude birth rates exist for each village.” However, it may not be a reason for the adjustment using the sampling weight. This study employed cluster sampling, under which it selected 80 and 40 villages first, then enumerated all the birth and neonatal death cases in the selected villages. Suppose the authors employed the simple random sampling method (without considering the probability-proportionate-to-sizes of villages). In that case, each case has a different probability of being selected in the sample due to differences in villages' population sizes. This point may be a reason for the adjustment for sampling weight in the estimate of neonatal mortality. | As the reviewer pointed out, in this study we have not employed the probability-proportionate-to-sizes for cluster sampling. What we intend to mention in the manuscript is the number of inhabitants in each village was used as the sampling weight, because the numbers of births were proportional to the numbers of inhabitants. We have modified the relevant part of the manuscript. | P8L129-131: We considered that each village is a cluster, and assumed that the crude birth rate in each village is the same. Therefore, the estimated number of inhabitants was used as the sampling weights for adjustment. |
| 2. The authors modified the Discussion section to highlight the importance of ensuring quality care, enhancing the referral system. Although they also modified the concluding statement (Lines 316-) to emphasize the importance of seeking solutions at the community level, this statement seems not reflect the issues of quality care and referral system. Since the Discussion section was reorganized based on the three-delay model, the concluding statement may also be structured according to the model. | Thank you for your comments.  We referred to the three-delays model to identify problems in the health care service, and classified the problems happening in the villages, in the referral system, and in the quality of care. This structure is similar to the model. However, we proposed four different approaches to solve the problems. Therefore, the following modifications were made in this revised manuscript.   - The order of paragraphs in the **Results** and Discussion sections was changed. - The **Discussion** section is reorganized to express our suggestions. | - **Results** - **Discussion** |

**Reviewer #2:**
I confirmed that the authors have addressed my previous suggestions. I have some minor comments as follows.

| **Comment** | **Response** | **Changed part in the manuscript (new Line)** |
| --- | --- | --- |
| 1. Page17 Line320 I did not understand what “at fixed points” means. Please rephrase it. | We used the term ‘fixed points’ in contrast with ‘mobile’ or ‘outreach’ services. We added the explanation: ‘fixed service points such as health centers’ | - **Abstract** - P19L331: at fixed service points such as health centers |
| 2. Page17 Line316-325. I acknowledge that the authors know the real situation in rural Cambodia very well and provided very important recommendations at the end of the paper. However, as a research paper, the conclusion can be described in more simple and specific manners, based on the study findings. | Thank you for your comment. We restructured our discussion part from the viewpoints of health system transformation and of community engagement. We believe that this modification connects the findings to the conclusions. | P19L327-334: the last paragraph in the **Discussion** section |
| 3. Please add footnotes for S3 Table, and describe why information on cases 1, 4, 9, 19, 22, 25, and 27 is written by Bold Italic characters. | We added the footnotes accordingly:  *Notes: Cases written by Bold Italic characters are presented as quotations in the main text.* | **Supporting Information S3 Table** |
