# Supplementary material for: "Why did our baby die soon after birth?"—Lessons on neonatal death in rural Cambodia from the perspective of caregivers
Source: PLoS One. 2021 Jun 7;16(6):e0252663. doi: 10.1371/journal.pone.0252663 (PMC8183999; doi:10.1371/journal.pone.0252663)
Supplement: Attachment — Submitted filename: Response to Reviewers.docx [file pone.0252663.s008.docx]

**PONE-D-20-32314RI
"Why did our baby die soon after birth?" – lessons on neonatal death in rural Cambodia from the perspective of caregivers.**

**Additional Editor Comments:**

In this revision, the manuscript was greatly improved, but it needs further revision in the discussion.

| **Comment** | **Point by point responses** | **Changed part in the manuscript (new Line)** |
| --- | --- | --- |
| 1. P15L244: The paragraph is well written but the authors do not show any data (evidence) of this study. As I mentioned previously, this is a place where the authors should discuss their own unique data, but they are missing here. | 1. Thank you for your comment.  We add one sentence after the topic sentence to show the data from our study. | P15/L246-247: Our study implicated that pregnant women did not aware of the signs of labor onset, which resulted in delivery at home without any assistance. |
| 2. P15L254: The same as above. No data are shown from this study. | 2. Thank you for your comment.  We add one sentence after the topic sentence to show the data from this study. | P16/L259-260: Our study found the unavailability of health-care professionals due to unfavorable environmental conditions, such as heavy weather. |
| 3. P16L268: This paragraph is good as the authors are discussing their own data. | 3. Thank you for your comment. | --- |
| 4. P17L295: This topic sentence is not clear. In the previous paragraphs, the specific contents of each intervention are clearly written, but this sentence is just focusing on how to ensure quality care and it does not seem to be a specific intervention. In P15L241, the fourth intervention is described as "development of human resources," but this phrase is missing here. | 4. Thank you for your comment.  1) We revised the topic sentence to show the specific intervention.  2) At the same time, we also changed the description in the first paragraph of **Discussion** (from ‘development of human resources’ to ‘improvement of knowledge and skill in health-care professionals’).  3) Furthermore, to show the relationships between our findings and proposed interventions. we add the **Supporting information S4 Figure**. | 1) P18/L300-301: The fourth intervention focuses on how to improve knowledge and skill in health-care professionals to ensure quality care.  2) P15/L241-242: improvement of knowledge and skill in health-care professionals  3) P15/L243-244: Cross-relationships between the problems and the possible interventions are described in **the Supporting information S4 Figure**. |

**Reviewer #1:** All the comments made in the previous round of the review process have been addressed in the current manuscript.

**Reviewer #2:** I confirmed that the authors have addressed all of my suggestions, and thank them for their hard work since the first submission. Congratulations. I believe that this manuscript will save more newborns in rural Cambodia.
